# Galileo Galilei: Science vs. faith

**DOI:** 10.21542/gcsp.2017.10

**Published:** 2017-06-30

**Authors:** Alberto Zanatta, Fabio Zampieri, Cristina Basso, Gaetano Thiene

**Affiliations:** Department of Cardiac, Thoracic and Vascular Sciences, University of Padua Medical School, Padua, Italy

## Introduction

Galileo Galilei (1564–1642), professor of mathematics at the University of Padua from 1592 to 1610, was a pillar in the history of our University and a symbol of freedom for research and teaching, well stated in the university motto “Universa Universis Patavina Libertas” (Total freedom in Padua, open to all the world).^1^ He invented the experimental method, based on evidence and calculation (“science is measure”)^2^ and was able, by using the telescope, to confirm the Copernican heliocentric theory, a challenge to the Bible. Bertrand Russell (1872–1970), in his book “The Problems of Philosophy” stated: “Almost everything that distinguishes modern world from earlier centuries is attributable to science, which achieved the most spectacular triumphs in the seventeenth century. Together with Harvey, Newton and Keplero, Galileo was a protagonist of this scientific revolution in the late Renaissance”.^3^

His life was a continuous struggle to defend science from the influence of religious prejudices. He was catholic, forced by the Inquisition to deny his views, and was condemned to home arrest for the rest of his life. Here is the history of his life, a pendulum between science and religious beliefs.

## The early period in Pisa

Galileo was born in Pisa from Giulia Ammannati (1538–1620) and Vincenzo Galilei (1520–1591), a musician, on February 15, 1564 (the same month and year when Michelangelo Buonarroti died). On November 5, 1581, he was registered in the faculty of Artists at the University of Pisa as a student of Medicine. In Florence in 1583 he met the mathematician Ostilio Ricci (1540–1603) and took up Mathematics, leaving Medicine, but never graduated. On 1589, he became Lecturer of Maths at the University of Pisa, with a salary of only 60 ducats/year. It was in 1590 that he carried out the famous experiment of falling spheres from the Tower of Pisa.

## The Patavian period

**Figure 1.**
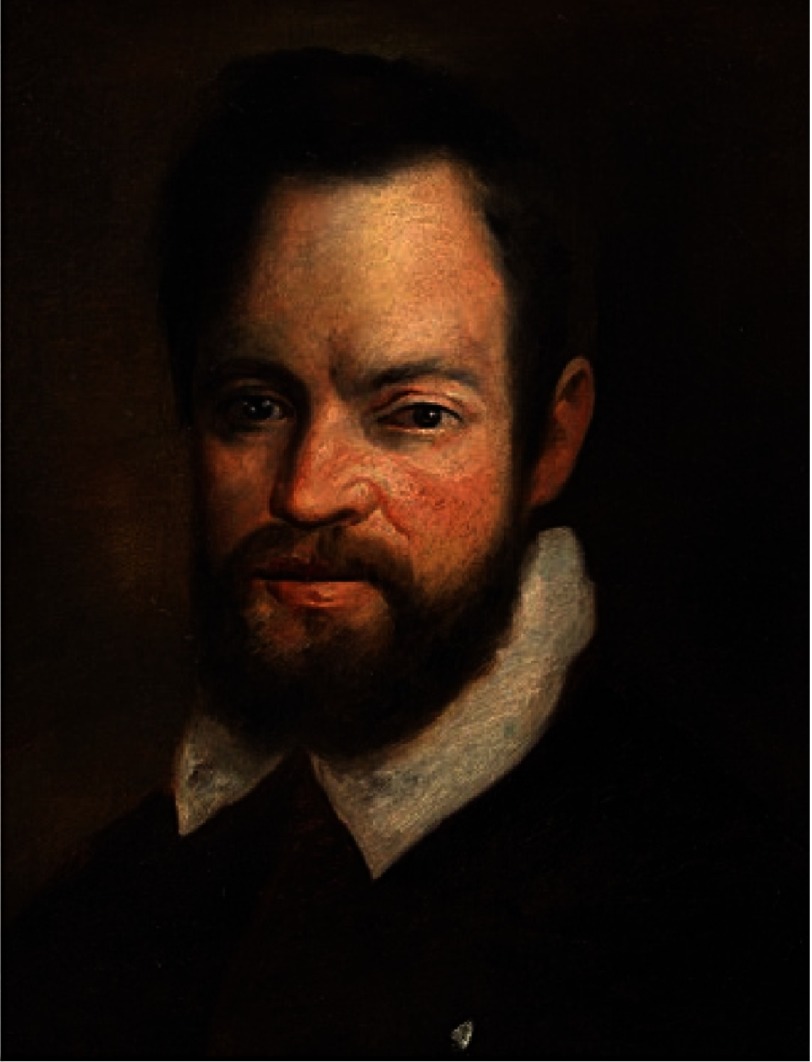
Portrait of the young Galileo by Domenico Cresti “Il Passignano”.

On September 25, 1592, at the early age of 28 years (Figure 1) he was appointed at the University of Padua to take over the vacant chair of Mathematics with a better salary (180 florins/year), still very little when compared to the salary of the anatomist and surgeon Fabricius ab Acquapendente (1533–1619) (1200 florins/year).

**Figure 2.**
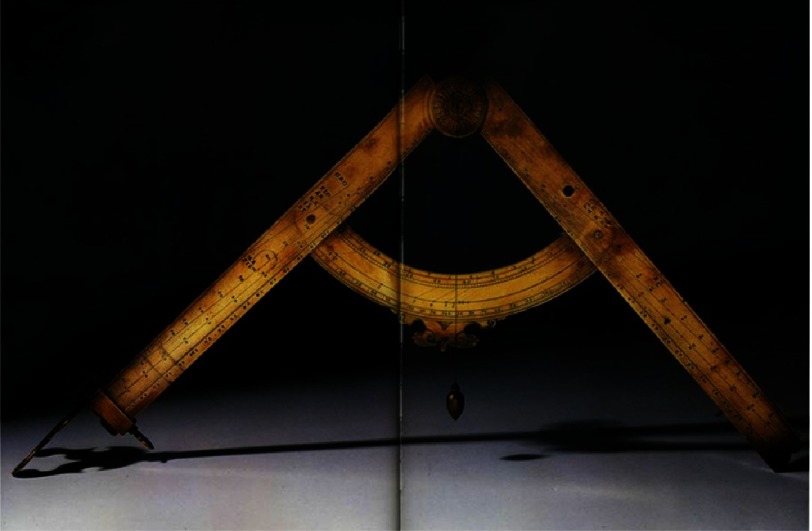
The geometric and military compass invented by Galileo.

In Padua, he spent 18 years, “the best years of my life”.^4^ He invented the compass (Figure 2), he wrote books on mechanics and cosmography for students. The teaching activity was hard, since the University council was very demanding.^5^

**Figure 3.**
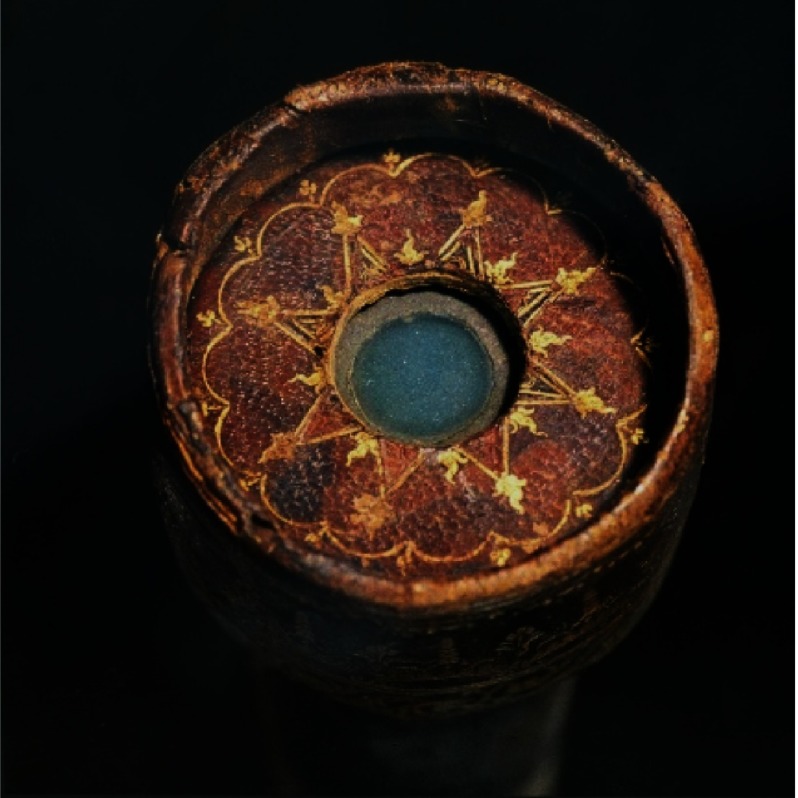
The “Eye” of Galileo’s telescope.

**Figure 4.**
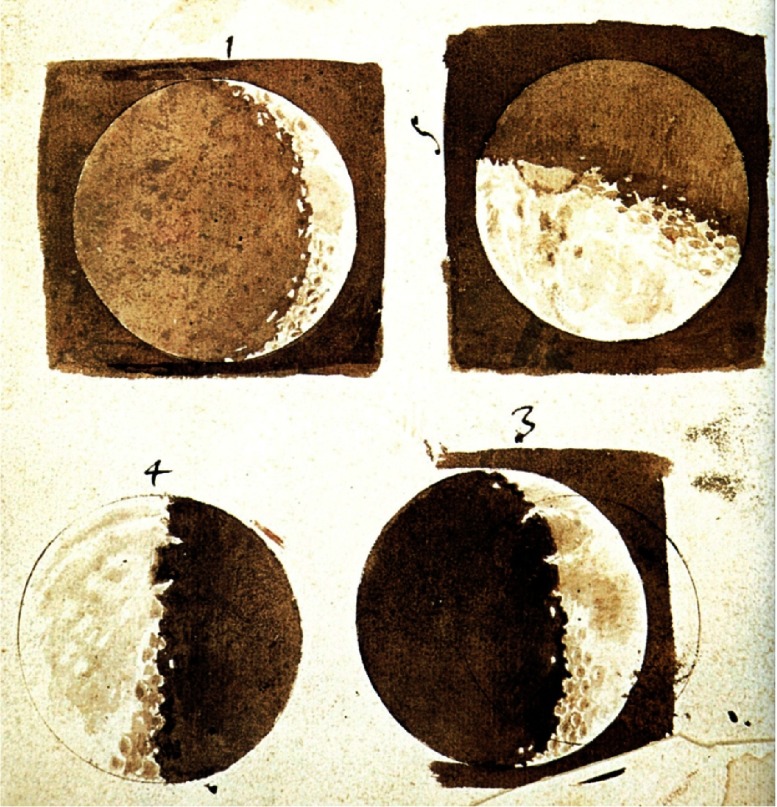
Drawings made by Galileo during his observations of the Moon.

He invented a telescope (Figure 3), with a 18–20 fold magnification,^6^ which made it possible to establish that the Milky Way consisted of a multitude of stars; to see the inequalities of the surface of the moon (Figure 4), the “rings” of Saturn, sun spots, the phases of Venus, and, last but not least, moons rotating around Jupiter, thus confirming the heliocentric theory of Nicolaus Copernicus (1473–1543).^7,8^

**Figure 5.**
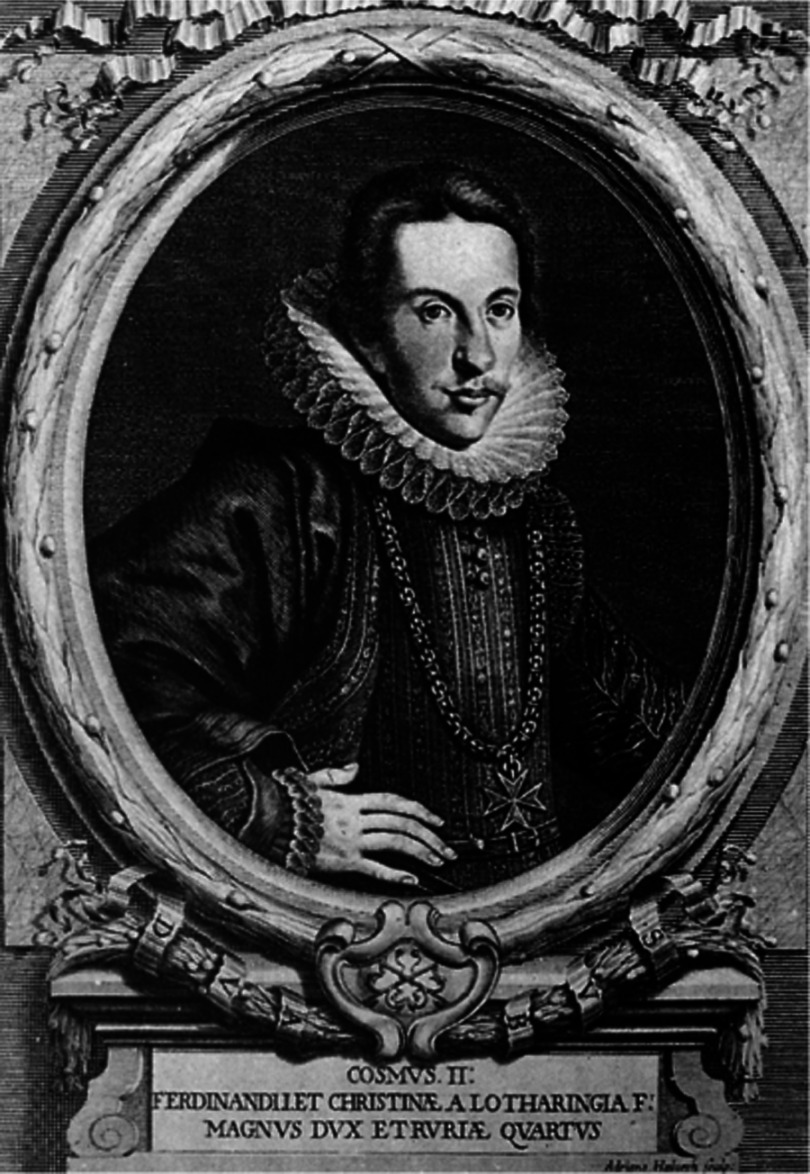
Cosimo II de’ Medici, Grand Duke of Tuscany.

The latter observation was made during the cold shiny nights of 7-13th January 1610, when he observed four objects moving around Jupiter, which he called “Medicea Siderea” (he was the tutor in Florence of the young Cosimo II de’ Medici (1590–1621) (Figure 5)). He wrote and published quickly the “Sidereus Nuncius” (“celestial announcement”), a book of only 55 pages, dedicated to Cosimo II, and full of notes and drawings proving his observations.^9^

On August 24, 1609, he had had the opportunity to present the telescope to the Doge Leonardo Donato and to the Council of Ten of the Serenissima Republic of Venice, watching at distance ships entering the Laguna from the top of the bell tower in St. Mark’s Square. They immediately realized the importance of the tool for military purposes and increased the salary of Galileo up to 1000 florins. Nevertheless, on June 10, 1610, he resigned from the chair in Padua, having accepted from Cosimo II de’ Medici the offer of a chair at the University of Pisa, free from teaching obligations and including Philosophy besides Maths.

On June 15, 1610, Galileo left Padua to move to Florence. His friend Giovanni Francesco Sagredo (1571–1620) wrote a letter to him, which was like a presentiment “Where you will find the freedom and monarchy of yourself you had in Venice?”^10^

The figure of Leonardo Donato (1536–1612), Doge in Venice from 1606-1610, deserves a note. He was the main supporter of Galileo at the time of his call to the University of Padua in 1592. In 1594, he was the one of the three reformers that had the rule of the University, and promoted, together with Fabricius ab Acquapendente and Paolo Sarpi (1552–1623), the building of the famous Anatomical Theatre, the first research laboratory in the history of Medicine.

Five years later William Harvey (1578–1657) arrived in Padua to study medicine and had the opportunity to dissect cadavers and to see valves in the veins. This observation inspired him to put forward the theory of blood circulation. At the time of Doge Leonardo Donato, the Republic of Venice was excommunicated (“Interdetto”) by the Pope Paolo V (Camillo Borghese (1552–1621)), for having condemned two criminal friars, instead of entrusting them to the church. Paolo Sarpi, a friar who defended the position of the Republic of Venice, was wounded during a subsequent assassination attempt.

Oddly enough, years earlier - in 1592 - Leonardo Donato was ambassador of the Venice Republic at the Vatican in Rome and met Camillo Borghese (the future Paul V), who threatened him by saying “If I will be Pope, at the first occasion I will excommunicate you” and Donato replied “If I will be Doge, I will not care of it”.

## The early Florentine period

On July 10, 1610 Galileo was nominated as “Primary Mathematician” at the University of Pisa and Philosopher by the Tuscan Grand Duke Cosimo II, with a salary of 1000 ducats/year (more than the salary offered by the Doge to remain in Padua). It is the period of triumph for Galileo, following the sidereal discoveries.

**Figure 6.**
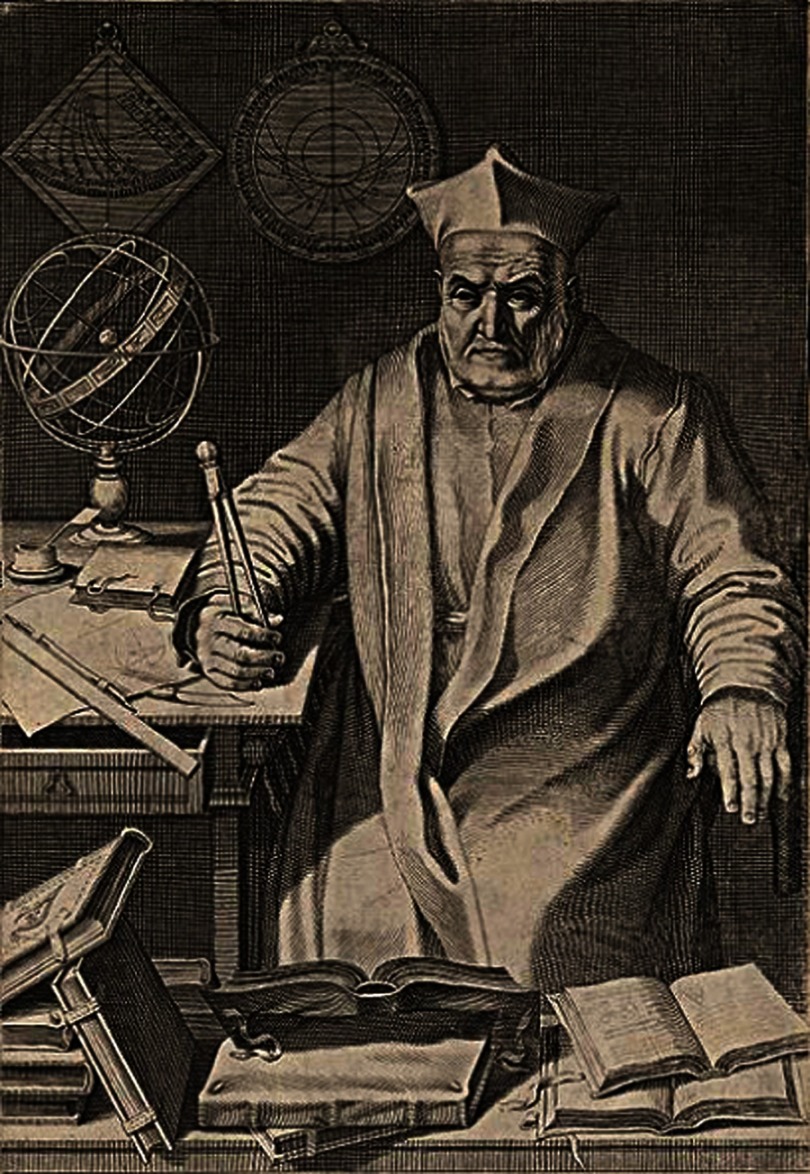
Christopher Clavius, Jesuit of the Roman College.

On April 1611, he visited Rome to meet Pope Paul V and the Roman College of Jesuits, the chief of which, Christopher Clavius (1538–1612) (Figure 6), fascinated by the observation of Jupiter’s moons, reserved him a friendly welcome.^11^

**Figure 7.**
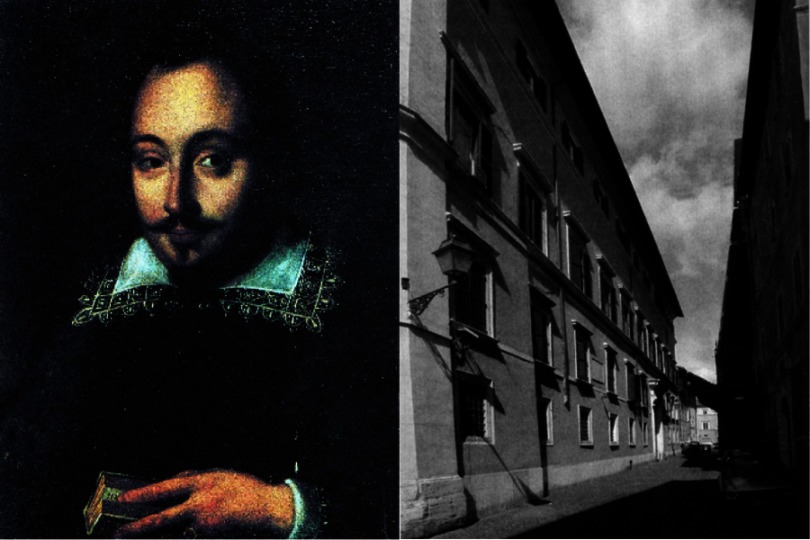
Federico Cesi, founder of the Lincei Academy in 1603.

On April 26, 1611 he was enrolled as a member of the Lincei Academy, founded in 1603 by the young Federico Cesi (1585–1630) (Figure 7), who named the telescope “Galileo spectacle”. Unfortunately, this honeymoon period soon ended.

The Dominican friars (“Domini canes” = the dogs of God) started attacking Galileo, because of the ideas behind Copernican theory, in which the sun, not the earth, was at the centre of the universe, thus contradicting the Bible. The Dominican friar Tommaso Caccini (1574–1648), on December 21, 1614, violently blamed Galileo from the pulpit of Santa Maria Novella in Florence, strongly defending the literal scriptures, and clearly accusing Galileo of heresy.

He said, “It is publicly well known that Mister Galileo keeps these two beliefs, namely the earth rotates around itself and the sun is motionless. These sentences reject the divine scriptures as dictated by the Holy Father and as consequence repel the faith, which teaches that we must consider true what the scriptures contain.”^11^

He referred to the book of Joshua 10, 12-15 where Joshua said:

*“Sun, stand still over Gibeon,and you, moon, over the Valley of Aijalon.So the sun stood still,and the moon stopped, till the nation avenged itself on its enemies”*.^12^

Moreover, in the Ecclesiastes, 1, 4 there is this sentence:

*“The earth is fixed and the sun arises on the east and sets on the west”.*^13^

The position of the Dominicans, who had the leadership of the Holy Office, was not universally shared by other representatives of the Church. Cardinal Cesare Baronio (1538–1607) in 1615 was quoted in a correspondence between Galileo and Cristina Lorena (1565–1637), mother of Archduke Cosimo II, as saying “The intention of the Holy Spirit is to teach how to go to heaven and not how the heaven is going”.

**Figure 8.**
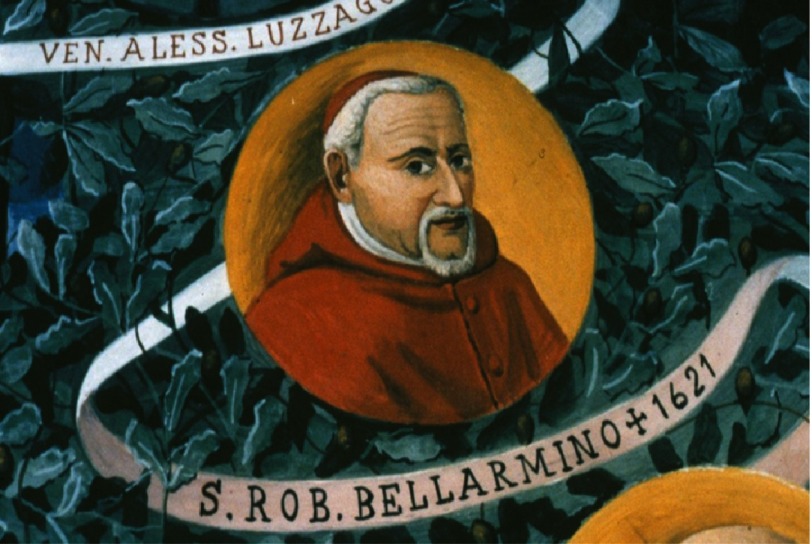
Cardinal Roberto Bellarmino graduated at the University of Padua.

Cardinal Roberto Bellarmino (1542–1621), a Jesuit, who had graduated at the University of Padua (Figure 8) and was the chief of the Holy Office (Inquisition), who had condemned Giordano Bruno (1548–1600) to the stake for heresy, because he questioned the trinity and believed in the existence of an infinite universe, wrote to Father Paolo Antionio Foscarini (1565–1616) on April 12, 1615: “Whenever the motionless of the sun and the movement of the earth around the sun will be proven, then we have to be prudent in interpreting the Scriptures which appear opposite and say that we do not understand rather than that is false what is demonstrated.”^14^

## The late Florentine period

On February 25, 1616, the Holy Office (Inquisition) officially condemned the Copernican Theory. Cardinal Bellarmino met Galileo and gave him a friendly warning not to sustain, teach, or refer to Copernican theory, otherwise the risk was to undergo Inquisition.^15^

On March 5, 1616, the book “De revolutionibus orbium coelestium” by Nicolaus Copernico was prohibited and the heliocentric theory was refused, being considered opposite to the Scriptures. Unfortunately, Cardinal Bellarmino and Cosimo II died in 1621, and with them Galileo lost his supporters.

**Figure 9.**
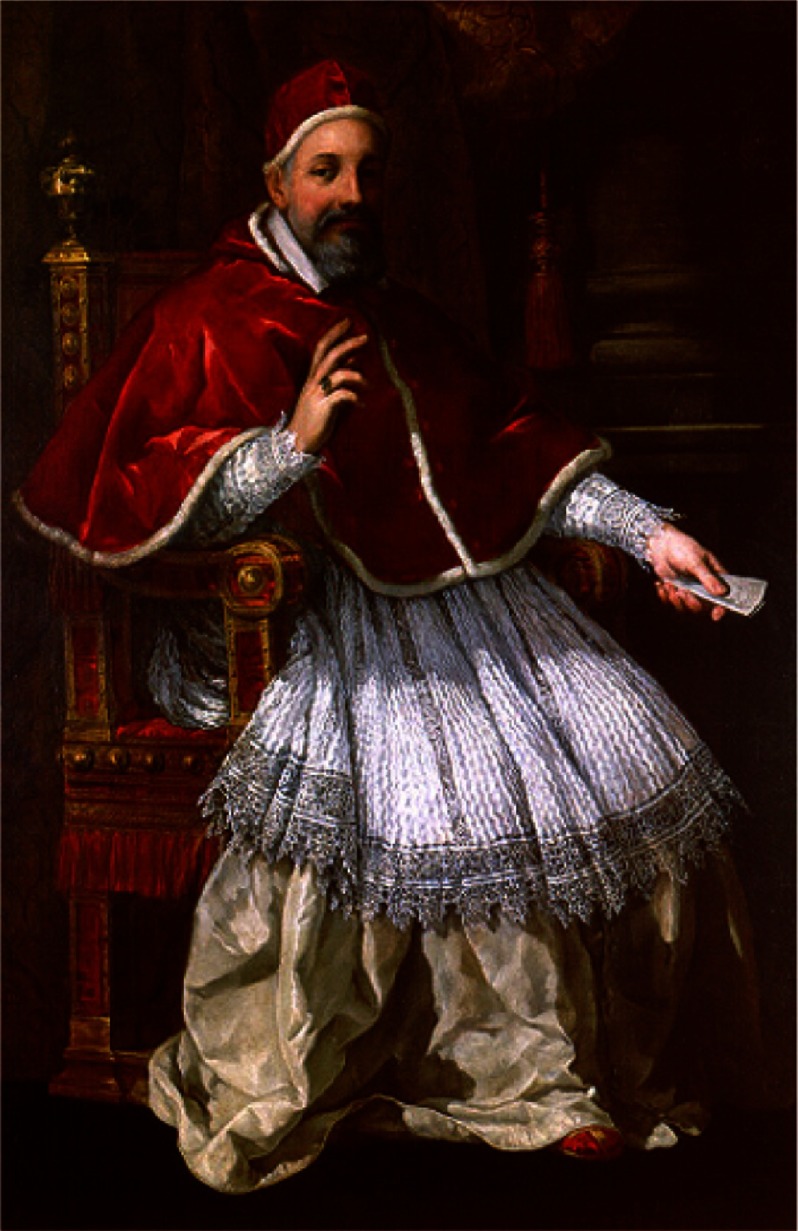
Maffeo Barberini, Pope Urban VIII.

**Figure 10.**
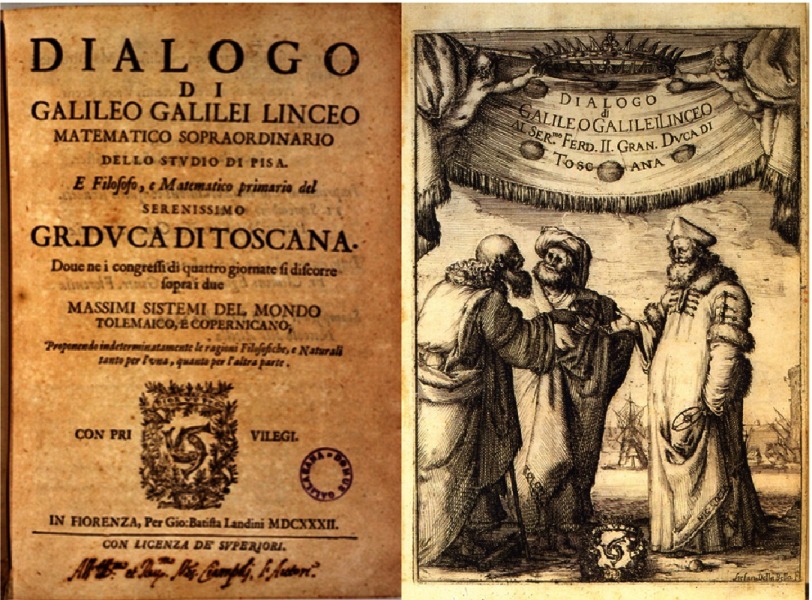
The “Dialogue concerning the Two Chief World Systems” published in 1632.

On August 6, 1623, Cardinal Maffeo Barberini (1568–1644) (Figure 9), adrmirer of Galileo, was elected Pope with the name of Urban VIII. Galileo believed that, with the new Pope as a friend, the atmosphere might change and he started writing the “Dialogue” (Figure 10), where his theses were reported. The book received the “nihil obstat” authorisation by the Holy Office in Florence, and was published in 1632. The language was not Latin, but Italian, thus allowing it to be read by lay people.

Unexpectedly, Pope Urban VIII changed his mind and submitted Galileo to the Inquisition for having published the book without his authorisation (“imprimatur”). Galileo was reluctant to undergo the trial, claiming health problems. Thereafter, the Pope sent a committee of physicians to Florence to check whether the impossibility of moving to Rome was true. He was found to be affected by “pulsus irregularis et inegualis” (atrial fibrillation?), kidney stones and melancholy. Nevertheless he was regarded healthy and forced to travel to Rome.

**Figure 11.**
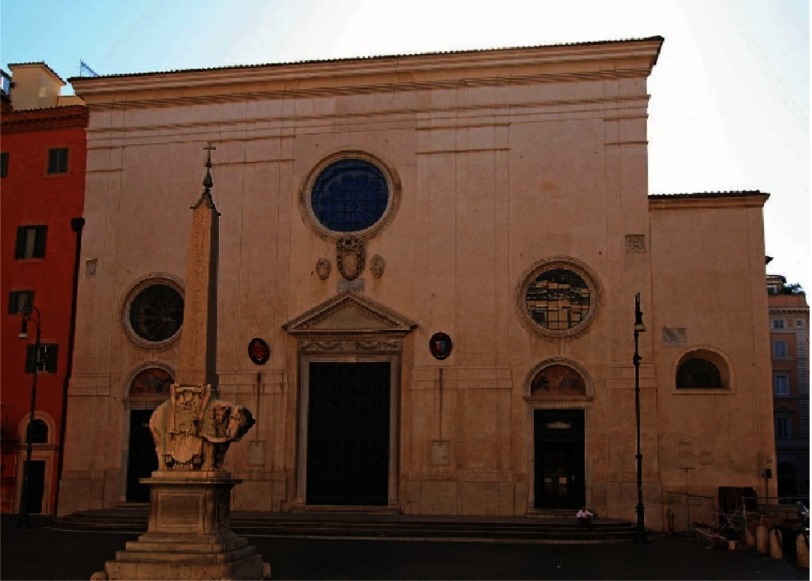
S. Maria Minerva church.

He was put into jail and in June 1633, brought to trial in the Church of Santa Maria Minerva (Figure 11).^16^ He was charged with heresy for believing false doctrines, contrary to Sacred and Divine Scriptures, namely that the sun and not the earth is the centre of the universe. He was condemned to deny, curse, and hate his mistakes (“abiura”). “With sincere feeling and faith I abjure, swear and abhor my mistakes and heresies contrary to Catholic and Apostolic Roman Church.”^17^

He was forced not to treat orally or to write any more on the issue, and condemned to home arrest for the rest of his life. The book was prohibited forever.

**Figure 12.**
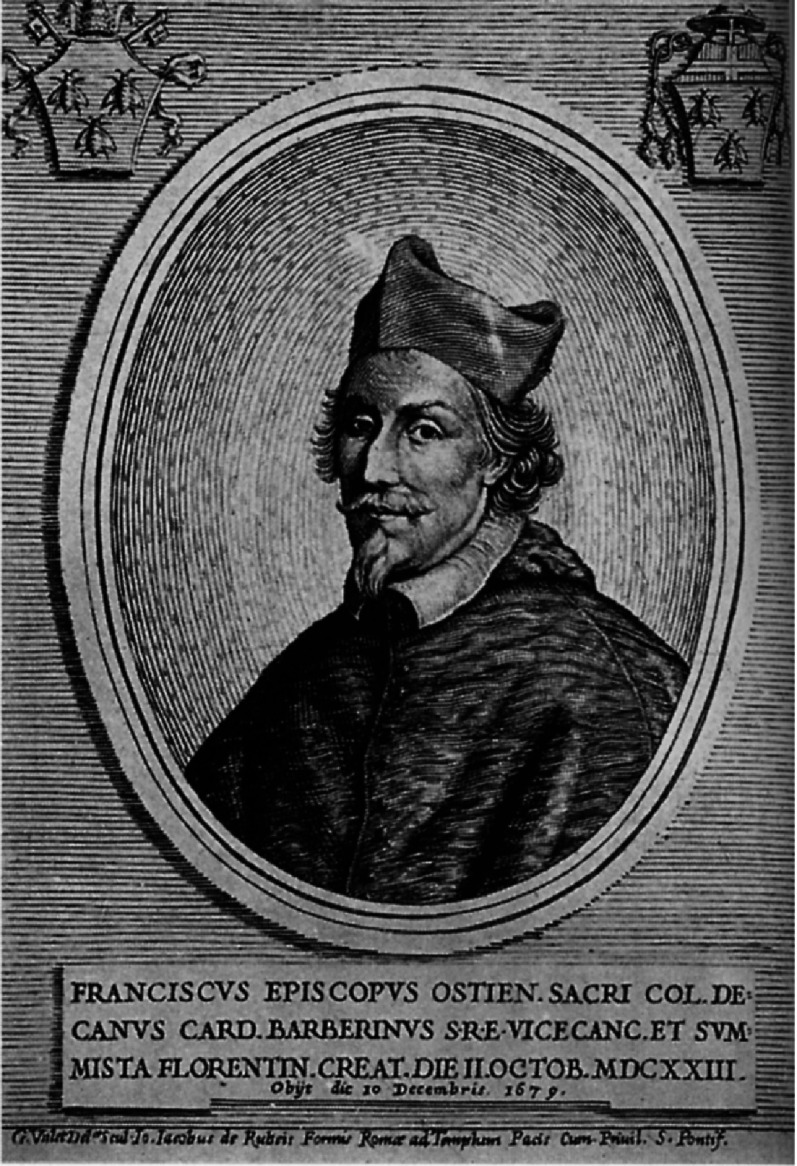
Cardinal Francesco Barberini.

The Committee of the Holy Office Inquisition was made up mostly of priests and friars of the Dominican and Theatine orders. The leader of the Holy Office was a Dominican, Father Vincenzo Maculan (1578–1667). The day when the sentence of condemnation was read, Cardinal Francesco Barberini (1597–1679) (Figure 12), friend of Galileo, nephew of Urban VIII, and member of the Holy Office, deserted the meeting. He disagreed and did not want to share this dreadful decision.

## The decline and fatal outcome

Galileo was confined to his house in Arcetri, not so far from the cloister where his daughter Virginia (1600–1634) (Sister Maria Celeste) was living and where she died prematurely in 1634.^18^

**Figure 13.**
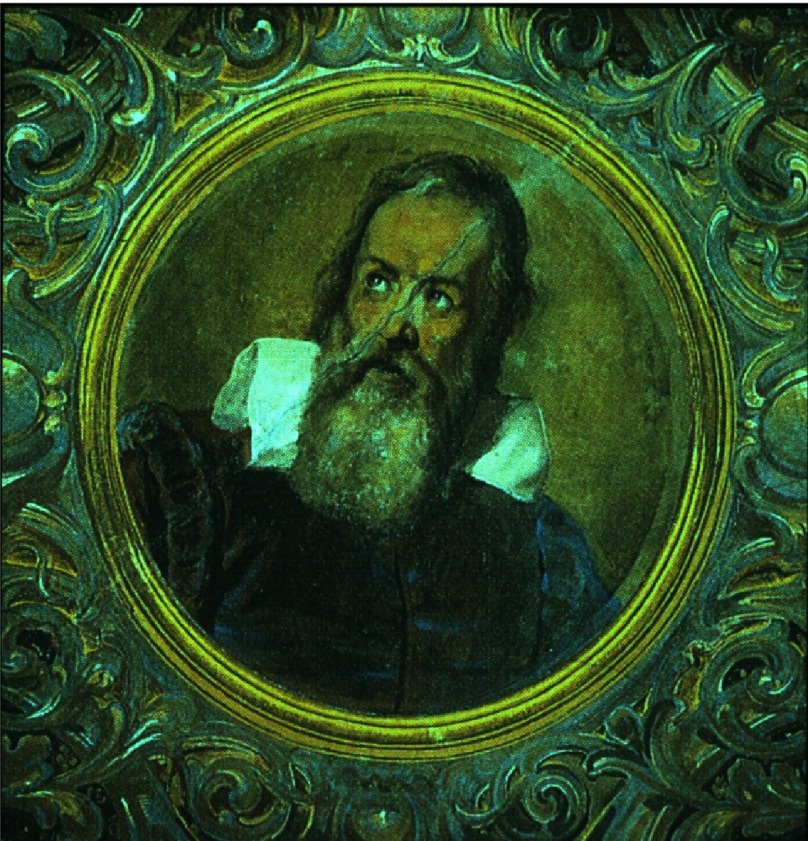
Galileo when he became blind bilaterally. (Painted on the ceiling of Aula Magna of the University of Padua, named Galileo Galilei in 1992).

In 1637 Galileo became blind bilaterally (Figure 13), most probably as a final complication of a long-standing reactive arthritis,^19–21^ and was assisted by his pupils Vincenzo Viviani (1622–1703) and Evangelista Torricelli (1608–1647).

In 1638 he published with Elzevier a new version of Dialogue, with a different title “Discourses and Mathematical Demonstrations Relating Two New Sciences”, confirming his views. On January 8, 1642, on the same day of his Jupiter observations by telescope, he died at Arcetri and was buried in a hidden Chapel of Santa Croce Church in Florence, without any official ceremony because he was still considered a heretic.

**Figure 14.**
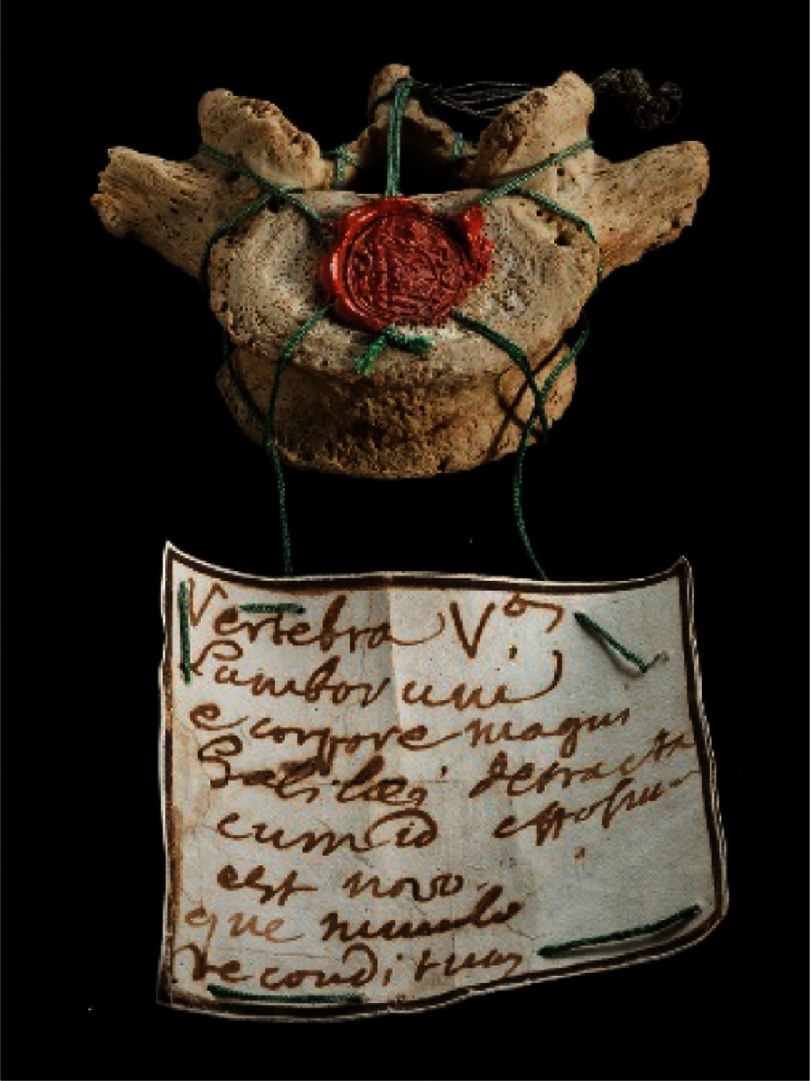
Galileo’s fifth lumbar vertebra preserved at the University of Padua.

In 1737, a mausoleum was built in the same church of Santa Croce and his remains were transferred together with those of his daughter Virginia. On that occasion, the exhumation gave the opportunity to remove the fifth lumbar vertebra (Figure 14), which is now on display at the University of Padua.^21^

## Four centuries later

The persecutions Galileo suffered during his life by the Roman Church were a source of longstanding controversy and diaspora between the Vatican and the University of Padua. Galileo was considered a victim of intolerance against science and intellectual freedom.

Four centuries later, the University of Padua celebrated a special year with meetings and *honoris causa* gradations. The Aula Magna (Great Hall) was named Galileo Galilei. Meanwhile Pope John Paul II (1920–2005) set up a committee of cardinals and theologists to re-examine the “Tolemaic-Copernican controversy of the XVI–XVII century”.

The committee, led by Cardinal Paul Poupard (1930 - ), worked hard for a few years and eventually gave a report to the pope, which is well summarised in its final part:

*“In this cultural historic context, far from our times, the judges of Galileo, unable to separate the faith from a millenary cosmology, believed, certainly wrongly, that the acceptance of the Copernican revolution, not yet definitively proven, could question the Catholic tradition and that therefore it was their duty to forbid its teaching. This subjective mistake of judgment, a clear-cut mistake nowadays for us, allowed them to adopt a disciplinary action of which Galileo “deeply suffered”. These injustices have to be honestly recognised, as you, Blessed Father, asked”.*^22^

The Rector Magnificus of the University of Padua, Prof. Mario Bonsembiante (1928–2009), invited the Pope to the final ceremony of the “Galileo Year 1992”. The Pope could not attend, however he sent a letter to the Rector in which he stated:

*“He attempted to contribute to the better knowledge of the truth, a purpose which is the common vocation of both scientists and theologists”.*^22^

This sentence was the best way to close the split between a lay university and the Roman Church.

The late Prof. Paolo Rossi (1923–2012) from Florence when he received the “laurea ad honorem” at the Padua University, noted: “Science has methods, aims and objectives, which are clearly different from the religious faith. It deals with knowledge, not with salvation. It tells how sky and earth go, but it does not help men to take decisions about their spiritual health and moral values”.^23^

As Kant said in his *Critique of Practical Reason*: “Two things fill the mind with ever-increasing wonder and awe, the more often and the more intensely the mind of thought is drawn to them: the starry heavens above me and the moral law within me”.^24^

Galileo suffered through the humiliation of having to deny his theories in order to save his life. He was Catholic, believed in God, but, on the other hand, he was a great believer in the role of science and the fascinating beauty of God’s creation.

After Galileo heard the sentence of condemnation, he had a final conversation with his supporter and friend, Malvasi^*^.^25^ *The Dialogue and its epilogue were the subject of a 1984 novel by Nicola Dalla Porta-Xydias, professor of Astronomy and successor of Galileo chair at the University of Padua.

Malvasi: God helps and blesses you, Maestro.

Galileo: What are you saying, God blesses me, a scientist?

Malvasi: God is nearer to you than to many others, you have encountered God today.

Galileo: In the humiliation, in the annihilation?

Malvasi: In the emptiness... Look for him and forget yourself. You will find him in the deep of your heart.

**Figure 15.**
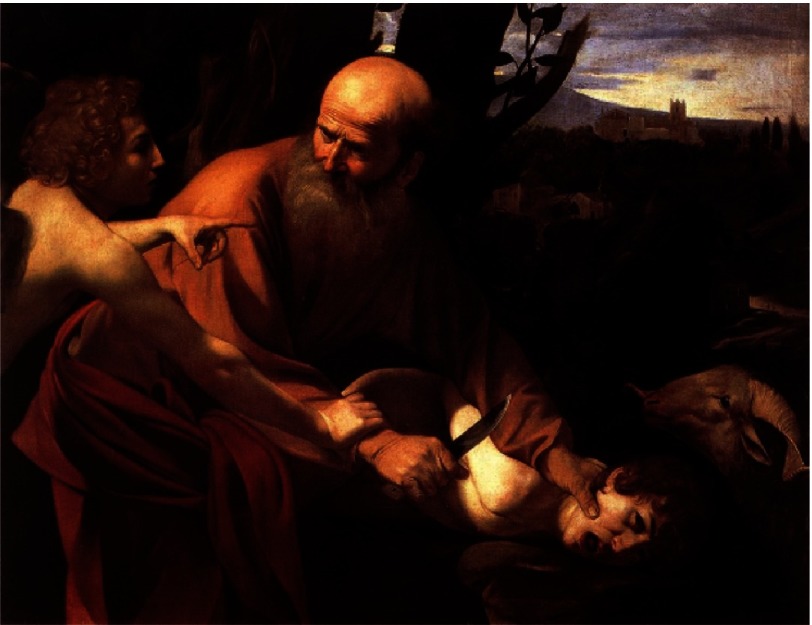
The sacrifice of Isaac. Caravaggio, 1603. Uffizi Gallery, Florence.

Could it be that God asked Galileo to leave aside the presumption of science, just as God asked Abraham to sacrifice his son Isaac to test his loyalty (Figure 15)?

## Conclusion and message to the young

The origin of science is an instance of struggle for intellectual liberty. When you read a book for your education and for improving your profession, look for the freedom and not only for the truth.
